# Postoperative Radiologic Changes in Early Recurrent Lumbar Foraminal Stenosis After Transforaminal Endoscopic Lumbar Foraminotomy for Lower Lumbar Segments

**DOI:** 10.3390/diagnostics15101299

**Published:** 2025-05-21

**Authors:** Chi-Ho Kim, Pius Kim, Chang-Il Ju, Jong-Hun Seo

**Affiliations:** Department of Neurosurgery, College of Medicine, Chosun University, Gwangju 61453, Republic of Korea; chiho13@naver.com (C.-H.K.); gamechanger@chosun.ac.kr (P.K.); jchangil@chosun.ac.kr (C.-I.J.)

**Keywords:** lumbar foraminal stenosis, endoscopic spine surgery, transforaminal endoscopic lumbar foraminotomy, restenosis, recurrent lumbar foraminal stenosis

## Abstract

**Background/Objectives:** One of the surgical treatments for lumbar foraminal stenosis, full endoscopic foraminotomy, is known for its numerous advantages and favourable clinical outcomes. While previous studies have analyzed preoperative radiological risk factors associated with recurrence within one year after endoscopic foraminal decompression, no research has investigated postoperative radiological changes. The aim of this study is to analyze the radiological changes occurring in cases of early recurrence within six months after endoscopic foraminal decompression. **Methods**: A retrospective review was conducted on patients with unilateral lumbar foraminal stenosis who underwent full endoscopic foraminotomy at a single institution. The study included 11 recurrent patients who initially experienced symptomatic improvement and sufficient neural decompression on radiological evaluation, but exhibited recurrent radicular pain and radiological restenosis within six months postoperatively. Additionally, 33 control patients with favourable clinical outcomes and no evidence of restenosis were analyzed. Preoperative and postoperative plain X-ray imaging was used to evaluate sagittal and coronal parameters reflecting spinal anatomical characteristics, including disc height, foraminal height, disc wedging, coronal Cobb’s angle, total lumbar lordosis angle, segmental lumbar lordosis angle, and dynamic segmental lumbar lordosis angle. The study aimed to analyze postoperative changes in these parameters between the recurrent and control groups. Clinical outcomes were assessed using the Visual Analog Scale (VAS). **Results:** There were no significant differences between the groups in terms of age, sex distribution, presence of adjacent segment disease, or existence of Grade 1 spondylolisthesis. Analysis of preoperative and postoperative radiological changes revealed that, in the recurrent group, disc height and foraminal height showed a significant decrease postoperatively, while disc wedging and the coronal Cobb’s angle demonstrated a significant increase. In contrast, the control group exhibited a significant postoperative increase in the total lumbar lordosis angle and segmental lumbar lordosis angle. **Conclusions**: Progressive worsening of disc wedging and the coronal Cobb’s angle, and reductions in disc and foraminal height, along with minimal improvement in lumbar lordosis following TELF, suggest the presence of irreversible preoperative degenerative changes. Careful radiologic assessment and close postoperative monitoring are essential to identify patients at risk of early recurrence.

## 1. Introduction

Lumbar foraminal stenosis (LFS) is a common degenerative spinal disorder predominantly affecting the elderly, typically involving the lower lumbar levels—most frequently L5/S1 and L4/5—as a result of age-related degenerative changes in the lumbar spine [1,2]. It is known to develop due to a combination of factors, including degenerated and herniated discs, resulting in reduced disc height and foraminal height, hypertrophy of the facet joints and ligaments, and the presence of syndesmophytes [1,3]. Due to these characteristics, LFS is associated with various degenerative spinal conditions, such as spondylolisthesis, spondylolysis, degenerative kyphotic and scoliotic deformities, and spinal stenosis. Consequently, nerve root compression often occurs not only in the foraminal zone, but also in the extraforaminal and hidden zones.

As the clinical characteristics of this condition have become widely known, the importance of surgical treatment for this disease has also increased. In the past, open surgeries such as microscopic foraminal expansion or fusion surgeries were primarily performed. However, over the past decade, advancements in minimally invasive equipment and techniques have led to the widespread adoption of endoscopic foraminal expansion surgeries. Transforaminal endoscopic lumbar foraminotomy (TELF), which utilizes Kambin’s triangle, can be beneficial in managing elderly patients who are at high risk under general anesthesia [4,5]. TELF has been reported to have shorter operation times, less bleeding, and favourable clinical outcomes and stability compared to microscopic foraminotomy [6,7,8]. Additionally, TELF has shown favourable clinical outcomes even for unilateral LFS accompanied by spondylolisthesis [9,10].

However, it remains difficult to assert whether long-term recurrence can be avoided and favourable clinical outcomes can be achieved without recurrence after TELF for LFS, and whether it can replace fusion surgery. Past studies have analyzed the clinical outcomes and recurrence factors of microscopic lumbar foraminal decompression [11,12]. Additionally, recent studies have analyzed risk factors associated with recurrence after TELF [13]. However, there have been no studies analyzing how radiologic factors change six months after surgery, compared to before surgery, in cases of recurrence within six months after TELF for LFS. This is the first study to analyze pre- and postoperative radiologic factors associated with early recurrence after endoscopic foraminotomy, with comparison to a control group. The purpose of this study is to identify which radiological factors show prominent changes before and after surgery in cases of early recurrence following TELF surgery.

## 2. Materials and Methods

### 2.1. Study Populations

Before commencing this study, IRB approval was obtained for the research design. This retrospective study was conducted at a single centre and targeted patients diagnosed with lumbar foraminal stenosis who underwent uniportal FELF for the condition from January 2020 to December 2022. Patients were selected according to the following inclusion criteria:

(1) Adults aged 18 years and older diagnosed with lumbar foraminal stenosis.
(2) Lumbar foraminal stenosis findings clearly observed on lumbar spine magnetic resonance imaging (MRI), with corresponding symptoms of lower extremity radiculopathy.
(3) Lower extremity radiculopathy persisting for more than 3 months, despite conservative treatment.
(4) Patients with confirmed moderate-to-severe foraminal stenosis at a single level on CT and MRI examinations, indicating compression of the nerve root [14,15].

The exclusion criteria were as follows:

(1) Concurrent spondylolytic spondylolisthesis, spondylolisthesis Grade 2 or higher, or segmental instability.
(2) Congenital foraminal stenosis.
(3) Concurrent severe Grade 3–4 central canal stenosis [16].
(4) Concurrent severe G2–3 lateral recess stenosis [14,15].
(5) Presence of other pathological conditions, such as infection, trauma, or tumours.
(6) Cases attributed to a herniated lumbar disc.

### 2.2. Selection of Case–Control Group

In this study, the definition and selection criteria for early restenosis after TELF in the case group were as follows:

(1) Improvement of lower extremity radiculopathy for at least 1 month post TELF.
(2) Recurrence of lower extremity radiculopathy at the same location as preoperative symptoms within 6 months post surgery.
(3) Radiological confirmation of recurrence of foraminal stenosis at the same site as the initial surgery.

The control group was selected based on the absence of recurrence of clinical symptoms and radiological restenosis for at least 1 year post TELF. To enhance the statistical power and significance of independent variables, a control group three times larger than the case group was collected.

### 2.3. Clinical Outcome Assessment

All basic clinical indicators, including age, gender, surgical level, and follow-up duration, were collected for all selected study subjects. Clinical outcomes were evaluated using the Visual Analog Scale (VAS) before surgery, post surgery, and at the 6-month follow-up.

### 2.4. Radiologic Assessment

All study subjects underwent radiological evaluations using static and dynamic simple radiographs before surgery, post surgery, and at the 6-month follow-up. The radiological factors assessed, and their respective measurement methods, were as follows [13,17]. To minimize inter-observer variability in radiographic measurements such as foraminal height and coronal Cobb’s angle, all parameters were independently assessed by two experienced spine surgeons. The mean of the two values was used for analysis, and any discrepancies beyond a predefined threshold (e.g., >3° or >2 mm) were resolved through consensus.

(1) Total lumbar lordosis angle (TLLA): The angle between the upper endplate of L1 and the upper endplate of S1.
(2) Segmental lumbar lordosis angle (SLLA): The angle between the upper endplate of the upper vertebra and the lower endplate of the lower vertebra, which comprise the surgical segment, provides information about the curvature of a specific segment of the lumbar spine.
(3) Coronal Cobb’s angle (CCA): The angle formed by the most tilted upper vertebra and the most tilted lower vertebra in an anteroposterior simple radiograph.
(4) Disc height (DH): The average height between the foremost and rearmost points of the intervertebral disc indicates the thickness of the intervertebral disc.
(5) Foraminal height (FH): The distance between the lower surface of the pedicle of the upper vertebra and the upper surface of the pedicle of the lower vertebra.
(6) Disc wedging (DW): The angle between the endplate adjacent to the upper part of the disc and the endplate adjacent to the lower part of the disc.
(7) Dynamic segmental lumbar lordosis angle (DSLLA): The difference in segmental lordotic angle (SLA) between flexion and extension postures provides information about the flexibility and range of motion of a specific segment of the lumbar spine.

### 2.5. Statistical Analysis

Statistical analyses were conducted using R version 4.2.2. All variables underwent appropriate descriptive statistical analysis. Statistical techniques were employed to compare groups as necessary. For the analysis of continuous data, the normality of each variable’s distribution was assessed, and either the Welch *t*-test or the Wilcoxon rank-sum test was applied accordingly. Categorical variables were analyzed using the Pearson chi-square test. A significance level of 0.05 was used to determine statistical significance.

### 2.6. Surgical Procedure

Before the surgery, intramuscular midazolam (0.05 mg/kg) and intravenous fentanyl (0.8 ug/kg) were administered, and the procedure was conducted under conscious sedation with transforaminal local or epidural anesthesia. An endoscopic system with an outer diameter of 6.3 mm, a working channel diameter of 3.7 mm, and an optical angle of 30° was utilized for the procedure.

All patients underwent surgery using the transforaminal endoscopic lumbar foraminotomy (TELF) technique. The surgical procedure involved targeting the SAP base as the landing point near Kambin’s triangle and advancing the working channel to this point. Utilizing endoscopic instruments and a burr, bone work was performed on the SAP and isthmus, and in cases requiring additional decompression of the ENR, a full-scale TELF was performed by extending the bone resection to include the pedicle of the upper vertebral body and the upper endplate of the lower vertebral body [18]. After removing bone work and soft tissue, in cases requiring decompression of the ventral portion of the ENR, the working channel was further advanced to retract the root, followed by RF annuloplasty or removal of the protruding disc and osteophytes [19,20,21]. However, disc material within the interbody space not directly associated with compression of nerve roots was not removed [13]. In cases of L5–S1 foraminal stenosis, where accessing the target landing point with the working channel is often challenging, this study employed an endoscopic burr to perform bone work on structures such as the transverse process of L5, the isthmus of L5, and the superior articular process of S1. This enabled precise positioning of the working channel and endoscope at the appropriate target landing point. To ensure sufficient foraminal decompression of the ENR, careful identification of the anatomical portions contributing to foraminal stenosis via preoperative imaging is essential. Efforts and techniques to minimize ENR irritation by the working channel or equipment are crucial during the performance of adequate foraminal decompression. Furthermore, to safely address circumferential moderate-to-severe foraminal stenosis while minimizing unnecessary stimulation of the ENR, the mobile outside-in technique was utilized [14,15,20,22].

## 3. Results

### 3.1. Patient Characteristics

The study included a total of 44 patients, with 11 in the case group and 33 in the control group. The mean age in the case group was 69.2 years, while in the control group, it was 66.0 years, with no significant differences in age and gender distribution between the groups. L4–5 was the most frequently operated level in both the patient and control groups. There were no significant differences between the groups in the presence of ASD or G1 spondylolisthesis. At 6 months postoperatively, the VAS score was significantly higher in the case group (5.7 ± 1.0 vs. 2.3 ± 0.9, *p* < 0.001). The mean time to recurrence in the case group was 6.1 ± 1.3 months, and the follow-up period was 26.0 ± 5.7 months in the case group and 20.5 ± 3.2 months in the control group (*p* = 0.02). Complications were one case of dysesthesia and one case of motor weakness in the case group, and three cases of dysesthesia and one case of motor weakness in the control group (Table 1).

### 3.2. Illustrative Case

**Figure 1:**
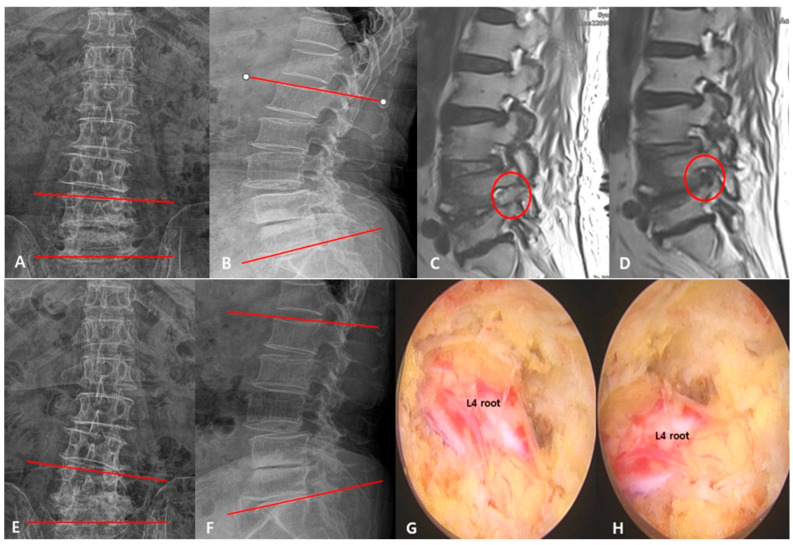
Illustrative case of 70-year-old female patient. (**A**,**B**) Preoperative simple radiograph demonstrating increased coronal Cobb’s angle and decreased lumbar lordosis angle (TLLA, SLLA). (**C**) Postoperative magnetic resonance imaging (MRI) showing widened left intervertebral foramen at L4/L5 level and sufficient decompression of nerve root. (**D**) Postoperative 6-month MRI showing recurred foraminal stenosis at L4/L5 level. (**E**,**F**) Postoperative simple radiograph showing increased coronal Cobb’s angle, worsened disc wedging, and little improvement in lumbar lordosis angle (TLLA, SLLA). (**G**,**H**) Intraoperative endoscopic view showing decompressed ENR (Lt. L4L root). (**A**,**E**) The red line represents the local coronal Cobb’s angle. (**B**,**F**) The red line represents the total lumbar lordosis angle. (**C**,**D**) The red circle marks the left intervertebral foramen.

A 70-year-old female patient, with no history of previous back surgery, presented with radiating pain in her left leg, originating from the back and outer thigh and extending to the front and outer side of the lower leg. Preoperative X-ray results revealed an increased coronal Cobb’s angle, a decrease in both total and segmental lumbar lordosis angles, and disc wedging on one side, leading to an overall reduction in disc height at the L4/5 level (Figure 1A,B). Postoperative MRI showed a widened left intervertebral foramen at the L4/5 level and sufficient decompression of the nerve root, but a postoperative 6-month MRI revealed a recurred foraminal stenosis at the L4/5 level (Figure 1C,D). Postoperative X-ray results revealed an increased coronal Cobb’s angle, worsened disc wedging, and little improvement in the lumbar lordosis angle (TLLA, SLLA) (Figure 1E,F). The intraoperative endoscopic view showed the decompressed ENR (Figure 1G,H).

### 3.3. Postoperative Radiologic Changes

In the case group, there were no significant changes in the TLLA, SLLA, or DSLLA at 6 months postoperatively. However, significant decreases were observed in DH (5.8 ± 2.4, *p* = 0.041) and FH (13.2 ± 1.2, *p* = 0.025), along with significant increases in DW (5.7 ± 1.5, *p* = 0.002) and CCA (12.2 ± 6.2, *p* = 0.041). On the other hand, in the control group, there were no significant changes in DH and FH, nor in DW and CCA. However, significant increases were observed in the TLLA (39.8 ± 10.2, *p =* 0.037) and SLLA (19.4 ± 4.5, *p* = 0.029), demonstrating statistically strong significance (Table 2).

## 4. Discussion

Surgical treatment for LFS can be broadly divided into foraminotomy and fusion surgery. As previously mentioned, LFS is often associated with degenerative spinal diseases, and historically, fusion surgery has been considered the gold standard, due to its ability to achieve neural decompression and correct degenerative changes simultaneously. However, in elderly patients or those with comorbid medical conditions, fusion surgery carries relatively high surgical risks. Additionally, fusion surgery leads to the loss of segmental motion, which can result in the degeneration of adjacent segments. Given the increasing human lifespan, the occurrence of adjacent segment disease after fusion surgery necessitates further extension of fusion procedures, making initial foraminotomy a preferable option when possible. For these reasons, the traditional microscopic foraminotomy via the Wiltse approach was commonly used in the past when planning non-fusion treatments. However, microscopic foraminotomy presents limitations in visualization and the operative field, making it challenging to perform neural decompression while minimizing facet violation. Consequently, postoperative dysesthesia and significant instability may occur during long-term follow-up. Several studies have reported that only about 80% of patients achieve good clinical outcomes with this approach [23,24,25,26,27].

TELF was developed to address the limitations of microscopic foraminotomy. Since Kambin and Sampson introduced percutaneous decompression for herniated lumbar discs, transforaminal techniques for foraminal decompression have significantly progressed. TELF enables deeper and less invasive access to the foraminal region than microscopic foraminotomy [28,29]. It facilitates exploration of both foraminal and extraforaminal zones [30,31], and has been effectively applied at the L5–S1 level using bone reamers [32,33]. With advances in endoscopic instruments and techniques, TELF has demonstrated safety and effectiveness in the treatment of LFS, with clinical improvement reported in approximately 85% of cases [7,8,34,35]. Recent reviews have shown that TELF provides better clinical outcomes than microscopic foraminotomy, with reported success rates of 85% and 80%, respectively.

However, TELF is a technically demanding procedure performed under local anesthesia with a transforaminal epidural block, involving relatively small incisions and small-diameter endoscopic instruments. Complications such as segmental artery injury, dorsal root ganglion (DRG) injury, motor weakness, incidental durotomy, instability, and the possibility of recurrence have been reported [19,36,37,38]. When performing TELF, the target landing point is set at the base of the SAP, near Kambin’s triangle, to decompress around the ENR. During this process, there is potential for irritation of the ENR, which can result in postoperative dysesthesia (POD). Kim et al. reported that the incidence of POD greater than Grade 1 after TELF is approximately 26%, which is relatively high [39]. While the complications associated with TELF are often related to the surgeon’s experience and technical issues, recurrence can occur even if TELF is performed perfectly. Early recurrent stenosis after TELF may necessitate fusion surgery, posing a significant concern for endoscopic surgeons. Investigating changes in radiographic factors during postoperative follow-up, in order to determine whether clinical outcomes will be favourable or whether there is a high likelihood of recurrence, is crucial. To date, several studies have analyzed preoperative radiographic risk factors associated with the recurrence of foraminal stenosis after microscopic or endoscopic foraminotomy. Haimoto et al. and Yamada et al. reported that significant preoperative degenerative lumbar scoliosis is associated with a higher likelihood of recurrence after microscopic foraminotomy [11,12]. Additionally, J.H. Seo et al. found that greater disc wedging, a larger coronal Cobb’s angle, and a smaller segmental lumbar lordosis angle on preoperative imaging are predictors of recurrence after TELF. They also reported that performing interbody discectomy could be a risk factor for recurrence following TELF [13]. However, to our knowledge, no study has investigated the factors associated with recurrence in routinely performed postoperative follow-up examinations, such as simple radiographs.

The current investigation showed that minimal improvement in the TLLA and SLLA following surgery is a predictor of restenosis after TELF. In contrast to the limited changes observed in the case group, both the TLLA and SLLA improved significantly in the control group. The preoperative loss of lumbar lordosis in the control group was likely caused by severe radicular pain, which restricted lumbar extension. Such pain-induced functional kyphosis is typically reversible with appropriate decompression and pain management. Restoration of lumbar lordosis postoperatively may positively affect overall spinal alignment, and could play a role in preventing recurrent foraminal stenosis. However, in certain patients, preoperative loss of lumbar lordosis may represent a fixed kyphotic deformity secondary to advanced disc degeneration, which is largely irreversible. Previous studies have demonstrated a strong association between lumbar disc degeneration and sagittal spinal imbalance, particularly the reduction of lumbar lordosis [40,41]. Moreover, biomechanical studies have shown that forward flexion of the lumbar spine increases intradiscal pressure, thereby potentially accelerating degenerative changes within the intervertebral discs. Microscopic foraminotomy has been reported to exacerbate coronal plane imbalance, potentially leading to progressive scoliosis and lateral segmental instability, while disc wedging has been identified as a potential contributing factor to recurrent stenosis [42].

In the present study, significant deterioration in DW and CCA was observed in the case group, along with a reduction in both FH and DH. These findings suggest the persistence of degenerative changes in both the sagittal and coronal planes following TELF. DW and CCA, in particular, serve as indicators of coronal segmental imbalance; their progression may reflect asymmetric disc collapse or evolving lateral instability, both of which may compromise neural decompression despite prior intervention. The combined progression of DW, FH, DH, and CCA may represent a key pathophysiological mechanism underlying restenosis after TELF. Among these parameters, DW and CCA are especially noteworthy, as they not only reflect localized disc degeneration, but also act as radiographic surrogates of coronal plane destabilization. Furthermore, the absence of postoperative improvement in SLLA and DSLLA may indicate pre-existing fixed sagittal imbalance, thereby diminishing the biomechanical efficacy of decompression and elevating the risk of recurrence. Notably, DSLLA reflects the dynamic mobility and functional alignment of the operative segment. A persistently reduced DSLLA may suggest segmental rigidity, potentially due to advanced disc degeneration or facet joint arthropathy, which could adversely affect long-term outcomes. In contrast, patients in the control group demonstrated improved DSLLA postoperatively, likely reflecting restoration of dynamic sagittal alignment, which may contribute to sustained foraminal patency during physiological loading.

In conclusion, reliable radiographic predictors of restenosis following TELF include not only static parameters, such as DW, CCA, and SLLA, but also dynamic metrics, like DSLLA. Comprehensive preoperative assessment and vigilant postoperative surveillance of these radiologic indicators may facilitate early identification of high-risk patients and inform individualized follow-up and intervention strategies.

### Limitations of the Study

Since early recurrence within 6 months after TELF for LFS is quite rare, the small number of cases in the patient group limits the statistical robustness of the findings. In addition, intra- and inter-observer reliability were not formally evaluated, which may limit the reproducibility of the radiographic measurements. Future research should involve prospective cohort studies with a larger sample size of early recurrence cases to provide more robust statistical evidence.

## 5. Conclusions

This study demonstrates that early recurrence after TELF is closely associated with ongoing degenerative changes in both sagittal and coronal spinal alignment. Significant decreases in DH and FH, along with worsening of DW and CCA, were observed in recurrent cases. Minimal improvements in lumbar lordosis further suggest the presence of irreversible preoperative imbalance. These findings highlight the need for careful radiologic evaluation of sagittal and coronal alignment before and after surgery. Patients with fixed kyphotic deformities or progressive scoliosis may be more prone to restenosis. Early detection of radiographic risk factors may aid in optimizing surgical strategies and long-term outcomes.

## Figures and Tables

**Table 1:** Demographic characteristics.

Variables		Cases (*n* = 11)	Controls (*n* = 33)	*p*-Value
Age (years)		69.2 ± 6.6	66.0 ± 7.5	0.412
Sex				
	Male	8 (72.7)	18 (54.5)	
	Female	3 (27.3)	15 (45.5)	0.286
Level of surgery				
	L3–4	3 (27.2)	7 (21.2)	
	L4–5	6 (54.5)	19 (59.4)	
	L5–S1	2 (18.2)	19 (21.2)	0.912
ASD				
	Present	1 (9.1)	4 (12.1)	
	Absent	10 (90.9)	29 (87.9)	0.750
Spondylolisthesis (GI)				
	Present	2 (18.2)	3 (9.1)	
	Absent	9 (81.8)	30 (90.9)	0.442
VAS				
	Preoperative	8.1 ± 1.4	7.5 ± 1.6	0.295
	Postoperative	3.3 ± 1.6	3.0 ± 1.7	0.574
	F/U at 6 months	5.7 ± 1.0	2.3 ± 0.9	<0.001 *
Recurrence time in months		6.1 ± 1.3		
F/U period (months)		26.0 ± 5.7	20.5 ± 3.2	0.02 *
Complications				
	Dysesthesia	1 (9.1)	3 (9.1)	1.0
	Motor weakness	1 (9.1)	1 (6.1)	0.712

Numeric values are depicted as averages with standard deviations, whereas categorical values are expressed as numerical figures (in rates or percentages). * *p* < 0.05, statistically significant differences.

**Table 2:** Comparison of postoperative radiological changes.

	Preoperative Values	6-Month Follow-Up Values	*p*-Value *
Cases (*n* = 11)			
Disc height	7.1 ± 2.3	5.8 ± 2.4	0.041*
Foraminal height	15.3 ± 1.5	13.2 ± 1.2	0.025 *
Disc wedging (angle)	3.5 ± 0.9	5.7 ± 1.5	0.002 *
Coronal Cobb’s angle	9.6 ± 5.4	12.2 ± 6.2	0.041 *
Total lumbar lordosis angle	35.1 ± 10.1	34.3 ± 10.8	0.241
Segmental lumbar lordosis angle	11.3 ± 5.5	10.4 ± 6.7	0.725
Dynamic segmental lumbar lordosis angle	9.9 ± 4.8	5.8 ± 4.3	0.944
Controls (*n* = 33)			
Disc height	8.2 ± 1.9	7.4 ± 1.8	0.192
Foraminal height	15.1 ± 2.0	14.8 ± 2.1	0.281
Disc wedging (angle)	0.6 ± 1.2	0.9 ± 1.0	0.618
Coronal Cobb’s angle	4.5 ± 2.6	4.1 ± 2.5	0.238
Total lumbar lordosis angle	36.5 ± 10.5	39.8 ± 10.2	0.037 *
Segmental lumbar lordosis angle	17.2 ± 4.8	19.4 ± 4.5	0.029 *
Dynamic segmental lumbar lordosis angle	7.2 ± 3.6	6.9 ± 3.5	0.612

Values are presented as means ± standard deviations. The continuous variables above were analyzed by applying either the Welch *t*-test or the Wilcoxon rank sum test. * A *p*-value of <0.05 was considered statistically significant.

## Data Availability

All data obtained and analyzed for this clinical study are available from the corresponding author upon reasonable request.
